# The Autophagy-Related Organelle Autophagoproteasome Is Suppressed within Ischemic Penumbra

**DOI:** 10.3390/ijms221910364

**Published:** 2021-09-26

**Authors:** Francesca Biagioni, Federica Mastroiacovo, Paola Lenzi, Stefano Puglisi-Allegra, Carla L. Busceti, Larisa Ryskalin, Rosangela Ferese, Domenico Bucci, Alessandro Frati, Ferdinando Nicoletti, Francesco Fornai

**Affiliations:** 1 I.R.C.C.S. Neuromed, Via Atinense 18, 86077 Pozzilli, Italy; francesca.biagioni@neuromed.it (F.B.); federica.mast@neuromed.it (F.M.); stefano.puglisiallegra@neuromed.it (S.P.-A.); carla.busceti@neuromed.it (C.L.B.); ferese.rosangela@gmail.com (R.F.); domenico.bucci@neuromed.it (D.B.); alessandro.frati@uniroma1.it (A.F.); ferdinandonicoletti@hotmail.com (F.N.); 2 Department of Translational Research and New Technologies in Medicine and Surgery, University of Pisa, Via Roma 55, 56126 Pisa, Italy; paola.lenzi@unipi.it (P.L.); larisa.ryskalin@unipi.it (L.R.); 3 Neurosurgery Division, Human Neurosciences Department, Sapienza University, 00135 Rome, Italy; 4 Department of Physiology and Pharmacology “V. Erspamer”, University Sapienza of Rome, 00185 Rome, Italy

**Keywords:** autophagy vacuoles, proteasome, LC3, P20S, heat shock protein 70 (HSP70), ultrastructural morphometry, stoichiometry, compartmentalization

## Abstract

The peri-infarct region, which surrounds the irreversible ischemic stroke area is named ischemic *penumbra*. This term emphasizes the borderline conditions for neurons placed within such a critical region. *Area penumbra* separates the ischemic core, where frank cell loss occurs, from the surrounding healthy brain tissue. Within such a brain region, nervous matter, and mostly neurons are impaired concerning metabolic conditions. The classic biochemical marker, which reliably marks *area penumbra* is the over-expression of the heat shock protein 70 (HSP70). However, other proteins related to cell clearing pathways are modified within *area penumbra*. Among these, autophagy proteins like LC3 increase in a way, which recapitulates Hsp70. In contrast, components, such as P20S, markedly decrease. Despite apparent discrepancies, the present study indicates remarkable overlapping between LC3 and P20S redistribution within *area penumbra*. In fact, the amount of both proteins is markedly reduced within vacuoles. Specifically, a massive loss of LC3 + P20S immuno-positive vacuoles (autophagoproteasomes) is reported here. This represents the most relevant sub-cellular alteration here described in cell clearing pathways within *area penumbra*. The functional significance of these findings remains to be determined and it will take a novel experimental stream to decipher the fine-tuning of such a phenomenon.

## 1. Introduction

The peri-infarct region, which surrounds the irreversible neuropathology following an ischemic stroke is named ischemic *penumbra*. This term provides an operational definition, which emphasizes the borderline conditions for neurons placed within such a critical region. *Area penumbra* is shaped like a belt, which separates the ischemic core, where frank cell loss occurs, from the surrounding healthy brain tissue. Within such a brain region, nervous matter, and mostly neurons are impaired concerning metabolic conditions. In this peri-infarct area, maturation phenomena take place early, which may lead either to delayed cell death or, conversely, to reversed metabolic impairment and restored neuronal function. For such a reason, the fine analysis of sub-cellular organelles and biochemical pathways, which operate within *area penumbra* is key to understanding the biology of ischemia-induced brain damage. In turn, this is key to planning beneficial therapeutic interventions to obtain a morpho-functional rescue of such a brain area. Depending on various concomitant phenomena, opposite outcomes may occur in this area, consisting either in a reduction or magnification of the infarct volume [1]. Thus, sub-cellular substrates within neurons placed within ischemic *penumbra* are analyzed in an effort to plan beneficial post-ischemic treatments to improve the amount of rescued brain tissue and the clinical outcome of ischemic events.

*Area penumbra* is originally identified by using magnetic resonance imaging (MRI) [2] using T2-weighted images as a moderately dark (*penumbra*) region, being the ischemic core frankly dark, while the surrounding healthy tissue appearing as intensely white. This corresponds to the etymological definition of the word “*penumbra*” (partial shade) as it appears in the peri-infarct region surrounding the infarct (shaded) area. The optimal planning of a beneficial modulation of *area penumbra* requires an in-depth knowledge of those biochemical pathways, which operate at this level. The classic biochemical marker, which reliably overlaps with MRI-defined “*area penumbra*” is the over-expression of a chaperone, heat shock protein 70 (HSP70) [3]. However, other proteins related to cell clearing pathways recently became relevant to mark *area penumbra*. Among these, proteins belonging to the autophagy machinery were probed [4,5]. In fact, autophagy-related proteins, such as LC3, increase within *area penumbra* although their significance remains controversial [4,5,6,7,8].

In keeping with cell clearing systems, *area penumbra* also features an altered expression of proteasome-related proteins [9]. In fact, both autophagy and proteasome are modified during brain ischemia, where an interplay may take place concerning a shift from autophagy to proteasome activity [10] or vice-versa [11]. This is related to compensatory events, which are still in the process to be functionally elucidated [12]. For instance, no study so far investigated the compartmentalization of proteasome markers within *area penumbra* and no evidence is available concerning the concomitant expression of LC3 and P20S within *area penumbra* when referring to intracellular compartmentalization within neurons. This is key especially when considering the role played by these proteins in neuronal survival, and considering recent data showing co-localization of LC3 and P20S within the same vacuoles [13,14,15]. This vacuolar co-localization characterizes a novel organelle, named autophagoproteasome, where classic autophagy clearance merges with proteasome enzymatic activity. This is expected to produce two effects: (i) an empowered clearing organelle where eventually proteasomal and lysosomal enzymes synergize; (ii) a proteasome degradation compartment. This latter effect is named proteophagy [13,14,15,16,17,18]. It is likely that both effects take place depending on the timing of the organelle. In vitro studies demonstrate that within the autophagoproteasome LC3 and P20S proteins co-exist. Moreover, the very same proteins co-immuno-precipitate along with p62, ubiquitin and autophagy/proteasome substrates [16], which suggests a strong binding between autophagy and proteasome components within the same organelle. This precipitate derived from LC3 and P20S positive vacuoles still own a significant proteasomal enzymatic activity [16]. This suggests that a high amount of autophagoproteasomes provide an empowered clearing compartment, where enhanced proteolysis of cell substrates takes place. This novel organelle provides a single metabolic unit for what was once viewed as two distinct clearing compartments. Thus, in the present study, a specific investigation of P20S and LC3 compartmentalization within vacuoles from neurons within *area penumbra* is carried out. This allows us to measure the co-localization of autophagy- and proteasome-related markers, as well as the occurrence of the autophagoproteasomes within *area penumbra,* compared with control tissue.

## 2. Results

### 2.1. The Approach and the Outcomes of Middle Cerebral Artery (MCA) Occlusion on Various Brain Regions to Be Analyzed at Light and Electron Microscopy

**Figure 1:**
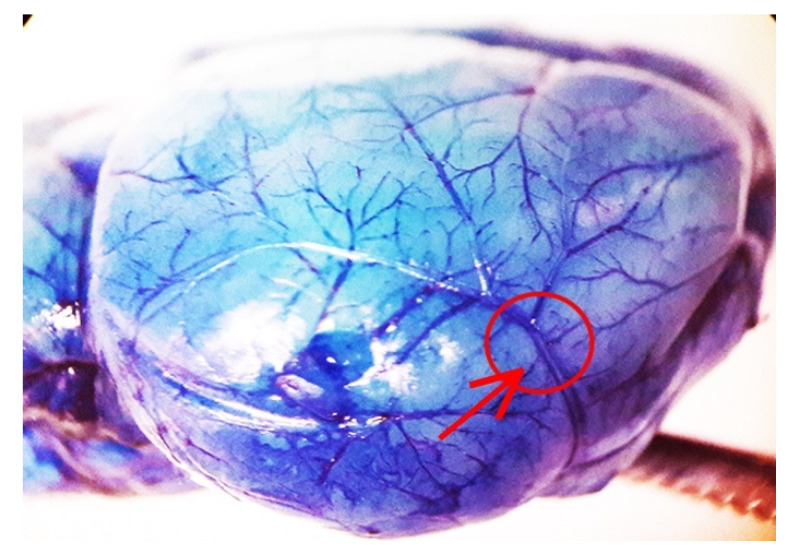
Representative image of electrocoagulation model of permanent focal ischemia obtained following Blue Evans perfusion of a sham-operated mouse.

**Figure 2:**
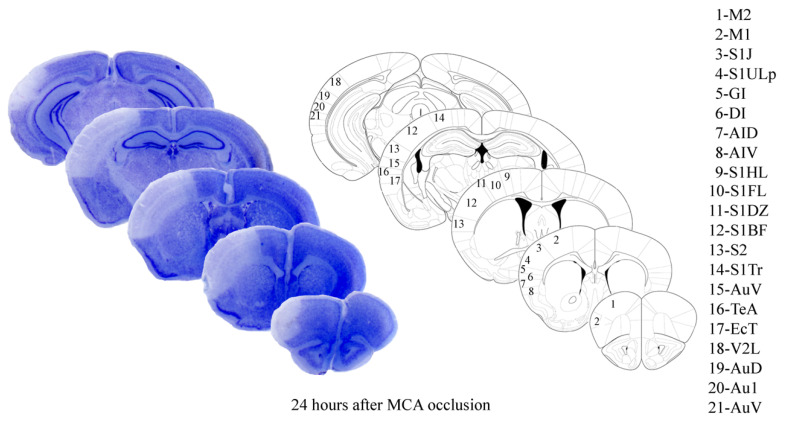
Nissl staining of representative mouse brain 24 h following right MCA occlusion and cortical regions involved in ischemic lesion. M2—secondary motor cortex; M1—primary motor cortex; S1J—primary somatosensory cortex, jax region; S1ULp—primary somatosensory cortex, upper lip region; GI—granular insular cortex; DI—dysgranular insular cortex; AID—agranular insular cortex, dorsal part; AIV—agranular insular cortex, ventral part; S1HL—primary somatosensory cortex, hindlimb region; S1FL—primary somatosensory cortex, forelimb region; S1D2—primary somatosensory cortex, dysgranular region; S1BF—primary somatosensory cortex, barrel field; S2—secondary somatosensory cortex; S1tr—primary somatosensory cortex, trunk region; AuV—secondary auditory cortex, ventral area; TeA—temporal association cortex; EcT—ectorhinal cortex; V2L—secondary visual cortex, lateral area; AuD—secondary auditory cortex, dorsal area; Au1—primary auditory cortex; AuV—secondary auditory cortex, ventral area.

When permanently occluding the distal, superficial aspect of MCA (Figure 1), the outcome of the ischemic damage, which is produced in C57Black mice is widespread (Figure 2). Its rostro-caudal extent recruits the primary motor cortex (M1, in the legend of Figure 2), the forelimb (S1FL, in the legend), dysgranular (S1DZ, in the legend) and upper lip (S1ULp, in the legend) regions of the primary somatosensory cortex, the primary somatosensory cortex (JAX region, S1J, in the legend), the secondary somatosensory cortex (S2, in the legend), and the granular/dysgranular insular cortex (GI and DI, respectively in the legend) reaching back the secondary visual cortex and the secondary as well as primary auditory cortex (V2L, AuD, Au1, and AuV, respectively, in the legend). The ischemic infarct was detectable as early as 2 h after MCA occlusion to reach the maximal size at 24 h. In detail, the infarct area, when evaluated by Nissl staining at 24 h after MCA occlusion corresponds to a rostro-caudal extent from bregma +2.34 back to bregma −3.28; the dorso-ventral extent varies depending on which rostro-caudal level is considered, being the maximum in the central core of the ischemic region. At this level, the dorso-ventral size of ischemia covers half of the brain.

This is carried out to show the model of permanent focal ischemia; the site of middle cerebral artery (MCA) occlusion corresponds to the centre of the red circle. This corresponds to the superficial course of the cortical extent of MCA after all perforant branches were sent off.

**Figure 3:**
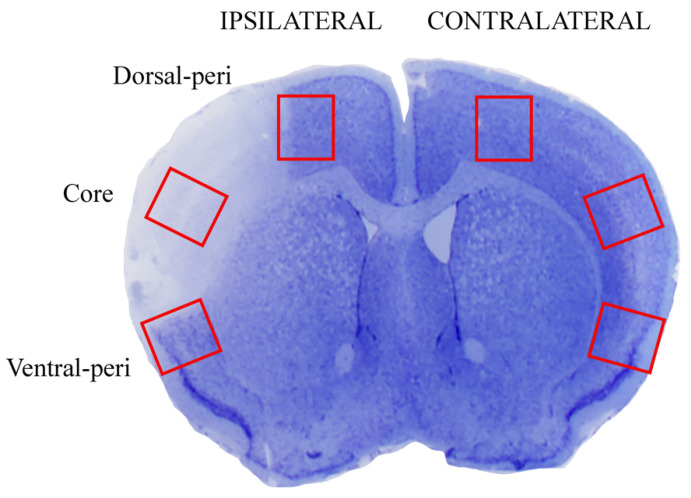
Representative picture of dissected regions.

**Figure 4:**
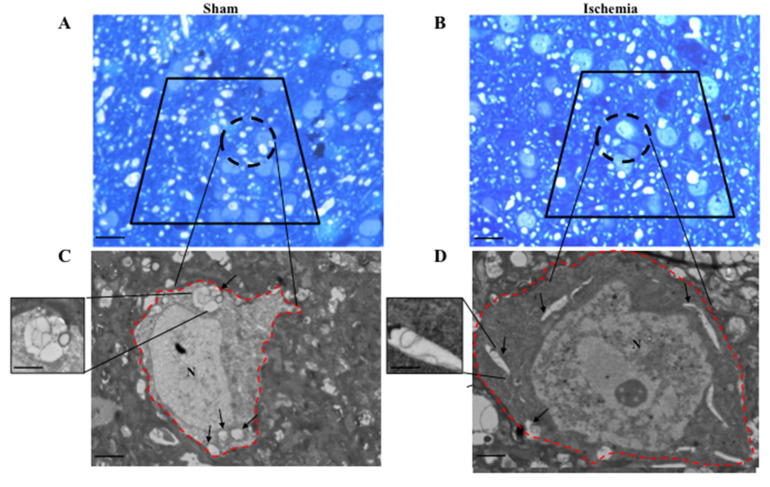
Representative semithin and ultrathin slices. Semithin slice of a brain area from a control sham-operated mouse (Sham) (**A**) and from the homologous brain area following ischemia representing *area penumbra* (Ischemia) (**B**). Slices were stained with toluidine blue to isolate neurons for transmission electron microscopy (TEM) as shown in (**C**,**D**) delineated by a red perimetral line. This encompasses authentic neurons from the control (sham) tissue and *area penumbra* (ischemia). At plain electron microscopy, neuronal alterations are evident, which characterize *area penumbra*. In detail, the cell body is swollen, the cytosol is more electron dense, a nuclear bleb is evident (asterisk), chromatin is disarranged. Arrows indicate vacuoles that occur within neurons. Scale bar = 60 μm (**A**,**B**); Scale bar = 0.3 μm (**C**,**D**); Scale bar (insert) = 8.3 μm. N = nucleus.

The counts within *area penumbra*, as shown in Figure 3, were carried out in the dorsal and ventral border of the central rostro-caudal extent (selected regions from 1.94 from bregma back to 0.14 from bregma). These were replicated in the counts carried out at light and electron-microscopy on corresponding regions. At this level, 4 slices spaced 550 μm each 10 μm thick were used for counting at light microscopy. For electron microscopy, the counts were focused within the same interval used for light microscopy, whereby using a matrix, we cut a 1 mm sliced region along the rostro-caudal axis to obtain 1 mm thick coronal slices each one further punched to dissect squared slices measuring 1 mm *×* 1.3 mm = 1.3 mm^2^ area placed at the dorsal and ventral border of the ischemic region. In this way, the further staining of each semithin section was already placed with *area penumbra* and the focus provided by the visualization of the semithin section allowed to further dissect an ultrathin area from *area penumbra* to be counted at electron microscopy. This is further evident in Figure 4A,B reporting representative semithin sections, showing how neurons from *area penumbra* as shown in Figure 3, are further identified for electron microscopy (Figure 4C,D). When evaluated at electron microscopy, ultrathin slices from *area penumbra* were compared with homologous regions from sham mice and homologous regions in the contralateral (non-ischemic) hemisphere. These multiple comparisons were carried out to avoid bias potentially produced by paracrine or systemic alterations triggered by the ischemic side. In fact, the expression of LC3 and P20S may depend on both focal and non-focal, even systemic, determinants. This may be triggered by a number of mechanisms, which would be relevant on the hemisphere contralateral to the ischemic region.

The term Dorsal-peri and Ventral-peri refer to the dorsal and ventral peri-infarct regions (respectively), both corresponding to *area penumbra* from the right ischemic (ipsilateral) hemisphere. Homologous regions are isolated from the left non-ischemic (contralateral) hemisphere. Slices were stained with thionin. When applying electron microscopy, additional control tissue was obtained from homologous regions from both hemispheres from sham-operated mice.

### 2.2. Effects of MCA Occlusion on HSP70 and LC3 Expression within Area Penumbra (General Features)

**Figure 5:**
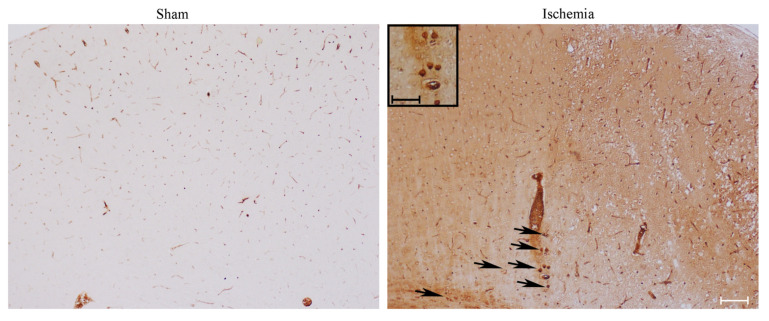
Representative HSP70 immuno-staining. This picture shows Heat Shock Protein 70 positive neurons stained with primary antibodies against inducible HSP70 within the *area penumbra* (Ischemia, in the representative picture) compared with the homologous area from a control mouse (Sham). Arrows point to HSP70 immuno-positive neurons. Scale bar = 100 μm; Scale bar (insert) = 50 μm.

**Figure 6:**
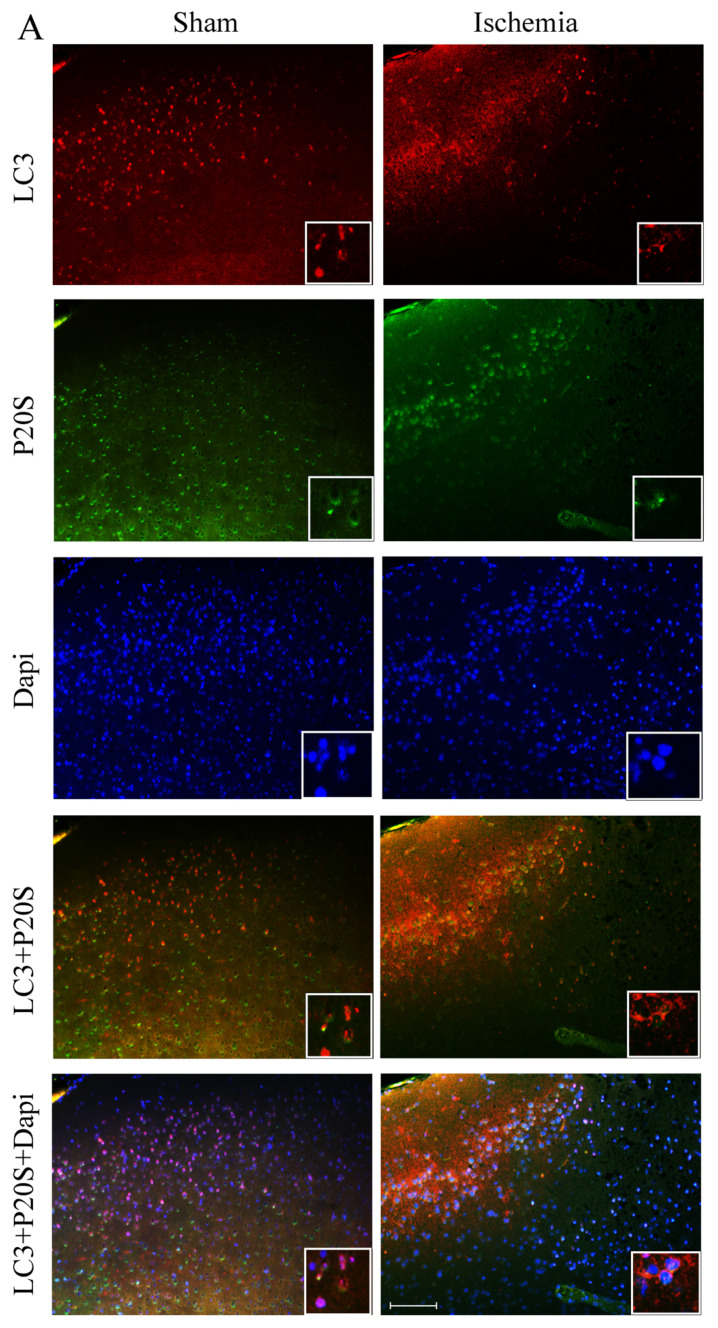

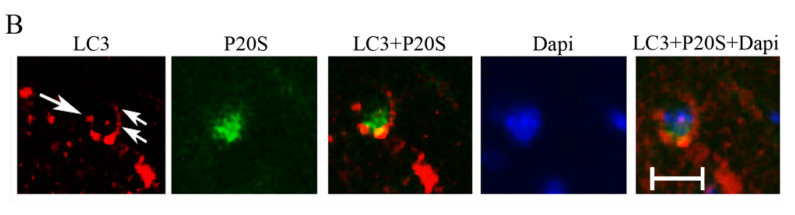
Representative immunofluorescence for LC3, P20S and Dapi. Pictures (**A**) report LC3-, and P20S-immunofluorescence along with Dapi-histofluorescence and their co-localization within *area penumbra* of a mouse following MCA (Ischemia) and the homologous region from a sham-operated mouse (Sham). It is evident the loss of LC3 + 20S co-localization, which occurs in *area penumbra* (ischemia) compared with control (Sham). (**B**) Representative images of cytosolic LC3 puncta occurring within neurons from *area penumbra*. White arrows indicate the merge between LC3 and P20S. Scale bar (**A**) = 100 μm; Scale bar (insert, **A**) = 10 μm. Scale bar (**B**) = 10 μm.

As shown in representative pictures of Figure 5, the classic marker of *area penumbra*, HSP70 marks the area to be stained for LC3 and P20S (Figure 6). In detail, by using primary antibodies, which stain specifically the inducible isoform of HSP70, the difference between a mouse undergoing ischemia and a sham control is remarkable (Figure 5). The staining is virtually absent in the sham mouse, while it roughly corresponds to a stained strip coursing along the peri-infarct region in the ischemic mouse. In the latter, both neuronal cell bodies and axons are stained (as evidenced by the arrows in Figure 5), along with blood vessels (the only structure which is stained in the sham mouse). The arrows indicate frankly immuno-positive neurons, which occur within *area penumbra*, while they are not evident and not present in the control (sham).

### 2.3. Effects of MCA Occlusion on LC3 and P20S Expression within Area Penumbra at Light Microscopy

**Figure 7:**
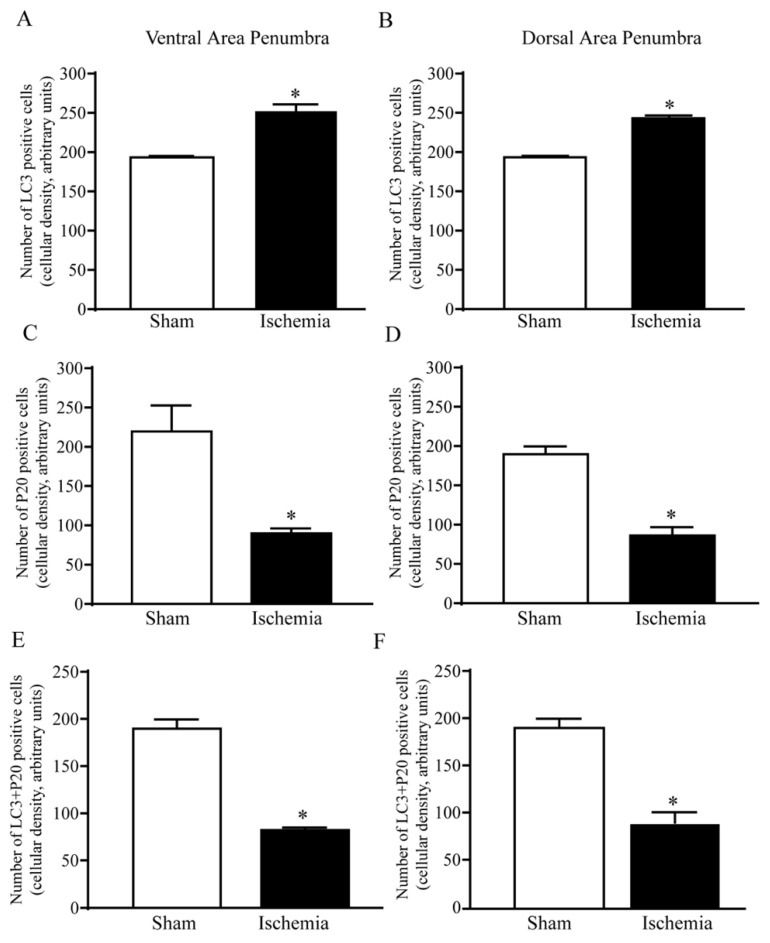
Quantitative analysis of immunofluorescence. Graphs report cellular density in the ventral and dorsal *area penumbra* (Ischemia) compared with homologous regions from sham-operated controls (Sham). Graphs report the mean ± S.E.M. of LC3-positive neurons (**A**,**B**), P20S-positive neurons (**C**,**D**) and co-localization of LC3 + P20S (**E**,**F**). * *p* ≤ 0.05 compared with controls (Sham). (**A**) df = 5, t = 4.660, *p* = 0.0055; (**B**) df = 5, t = 10.88, *p* = 0.0001; (**C**) df = 5, t = 5.586, *p* = 0.0025; (**D**) df = 5, t = 5.204, *p* = 0.0035; (**E**) df = 5, t = 12.12, *p* < 0.0001; (**F**) df = 5, t = 6.478, *p* = 0.0013.

The amount of immunofluorescence for LC3 and P20S is reported in representative Figure 6 along with DAPI-fluorescence in both a sham and ischemic mouse. In detail, DAPI was reduced in the peri-infarct region, which witnesses some cell loss within ischemic penumbra as reported and counted in Supplementary Figures S1 and S2. Despite a lower cell number, immunofluorescence for LC3 increases robustly within *area penumbra*, while the amount of P20S is dramatically reduced compared with the homologous region from a sham mouse (Figure 6). When counting immunofluorescent cells (graphs of Figure 7), LC3-positive cell density within ventral *area penumbra* increases roughly 1.5-fold compared with the sham homologous region (Figure 7A and Supplementary Figure S3). The increase in LC3 within the dorsal *area penumbra* is slighter but still significant (Figure 7B). In striking contrast, the density of P20S-positive cells is dramatically suppressed within *area penumbra* compared with the sham homologous area. Again, such an effect is greater within ventral (Figure 7C) compared with dorsal penumbra regions (Figure 7D). In the course of a preliminary search for a co-localization of LC3 and P20S within *area penumbra*, we could document a decrease in LC3 + P20S co-localization, which was even more pronounced compared with the decrease in P20S both within ventral (Figure 7E) and dorsal (Figure 7F) regions. The severe suppression of LC3 + P20S staining, which was surpassing at large the decrease in P20S was unexpected based on data obtained for every single antigen. In fact, if P20S was decreased within *area penumbra*, the concomitant increase in LC3 was supposed to attenuate the loss in the combined staining. This is expected based on the stochastic combination of LC3 and P20S since combined placement should not be further reduced when the decrease of one antigen (P20S) is concomitant with an increase in the other (LC3). Thus, a further decrease in LC3*P20S immunostaining cannot be the consequence of their stochastic combination. Therefore, within the ischemic HSP70-positive *area penumbra* something else occurs, which leads to a specific loss of autophagoproteasome (merging of LC3 with P20S). This represents a major issue of the manuscript, and it is specifically investigated in the second part of the present study by ultrastructural morphometry and quantitative stoichiometry. Data obtained at light microscopy already indicate that the suppression of merging between LC3 and P20S cannot be considered to depend solely on a decrease of P20S, since the concomitant increase in LC3 should have mitigated the loss of merging rather than worsening the merging of LC3 and P20S. The outcome of this first piece of investigation leads to some ad interim conclusions: (i) within cells from *area penumbra* there is an increase in LC3 which is concomitant with a decrease in P20S; (ii) within cells from *area penumbra* the increase in LC3 is reminiscent of the strip-like area stained by HSP70 and the same is true for the decrease in P20S, which suggests two additional markers to stain *area penumbra*; (iii) within *area penumbra* there is a suppression in the merging between P20S and LC3, which surpasses the decreases in P20S and it remains unexpected when considering the concomitant increase in LC3.

### 2.4. Effects of MCA Occlusion on the Amount of LC3, P20S and HSP70 within Area Penumbra at Western Blotting

**Figure 8:**
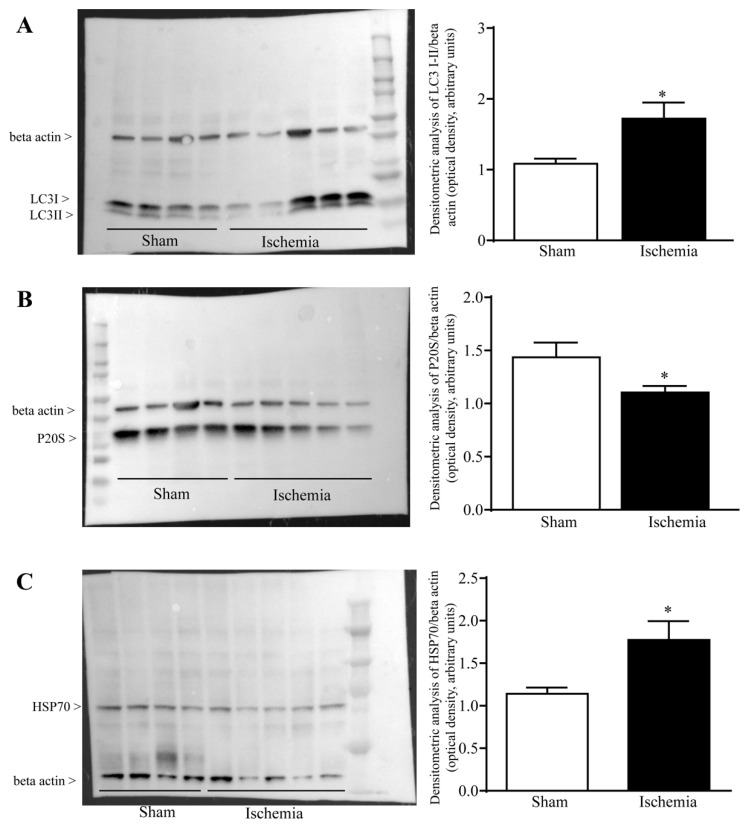
Modulation of LC3, P20S and HSP70 proteins following ischemia. Representative Western Blotting for LC3 (**A**), P20S (**B**), HSP70 (**C**) and beta actin within the *area penumbra* (Ischemia, in the representative picture) compared with the homologous area from a control mouse (Sham). Each graph reports the semi-quantitative densitometric analysis of each antigen expressed compared with the house-keeping protein beta actin. (**A**) df = 7, t = 2.396, *p* = 0.0478; (**B**) df = 7, t = 2.367, *p* = 0.0498; (**C**) df = 7, t = 2.433, *p* = 0.0452, * *p* ≤ 0.05 compared with Sham.

The amount of LC3 and P20S reported following immunofluorescence was confirmed by Western Blotting (Figure 8). This provides a confirmation that within the peri-infarct ischemic region defined *area penumbra*, there is a marked increase in LC3 (Figure 8A) with a concomitant decrease in P20S (Figure 8B). This is measured within homogenates from brain areas where HSP70 is increased (Figure 8C, which occurs by definition within *area penumbra*). These findings were further confirmed by using ultrastructural stoichiometry.

### 2.5. Effects of MCA Occlusion within Area Penumbra at Electron Microscopy (General Features)

**Figure 9:**
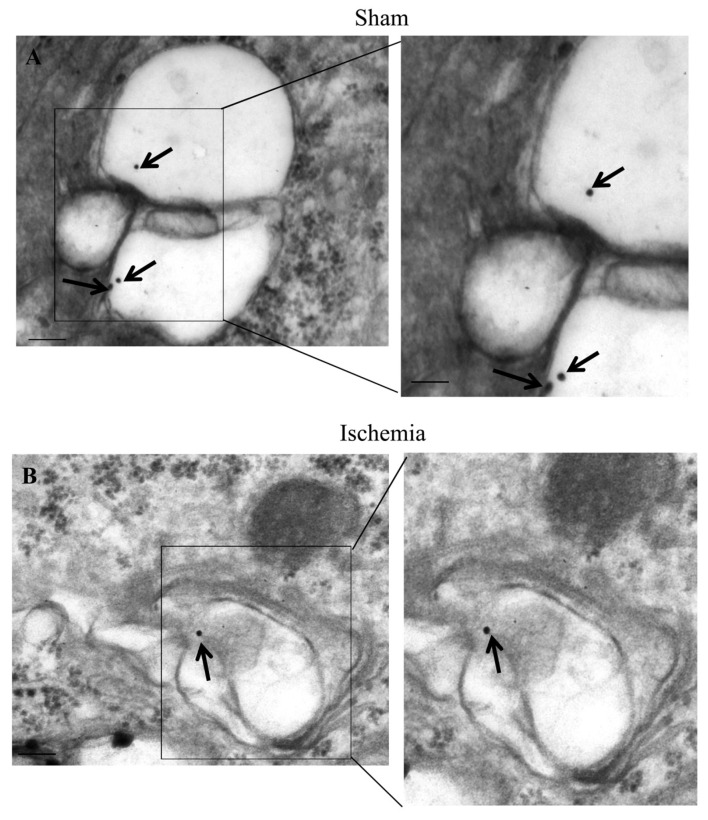
LC3 within *area penumbra*. Representative TEM micrographs showing LC3-positive vacuoles from an allo-cortical neuron from a ventral region homologous to *area penumbra* from a sham mouse (Sham, **A**) and from an allo-cortical neuron from ventral *area penumbra* from an ischemic mouse (Ischemia, **B**). Arrows point to LC3 immuno-gold particles (20 nm) within vacuoles. The insert highlights the immuno-gold particles within vacuoles. Scale bar = 0.2 μm (**A**,**B**), 0.1 μm (inserts).

**Figure 10:**
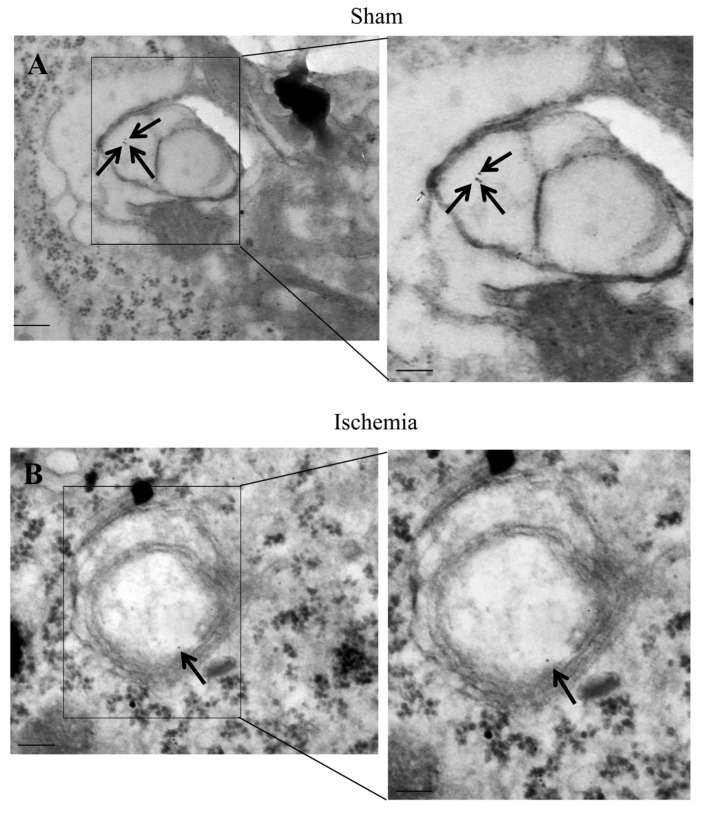
P20S within *area penumbra*. Representative TEM micrographs showing P20S-positive vacuoles within an allo-cortical neuron from a ventral region homologous to *area penumbra* from a sham mouse (Sham, **A**) and an allo-cortical neuron from ventral *area penumbra* from an ischemic mouse (Ischemia, **B**). Arrows point to P20S immuno-gold particles (10 nm) within vacuoles. The insert highlights the immuno-gold particles within vacuoles. Scale bar = 0.2 μm (**A**,**B**), 0.1 μm (inserts).

**Figure 11:**
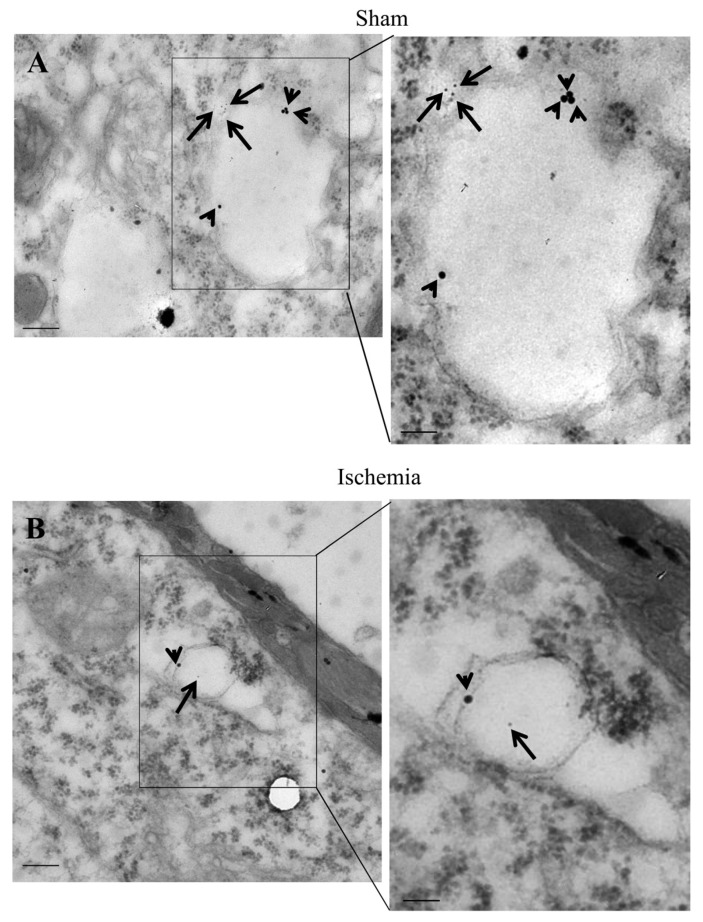
LC3 + P20S co-immuno-stained vacuoles. Representative TEM micrographs showing LC3 + P20S co-immuno-stained vacuoles within a cortical neuron from a ventral region homologous to *area penumbra* from a sham-operated mouse (Sham, **A**) and a cortical neuron from ventral *area penumbra* from an ischemic mouse (Ischemia, **B**). Arrowheads point to LC3 (20 nm) and arrows point to P20S (10 nm) immuno-gold particles within vacuoles. The insert highlights the LC3 + P20S immuno-gold particles within a vacuole. Scale bar = 0.2 μm (**A**,**B**), 0.1 μm (inserts).

**Figure 12:**
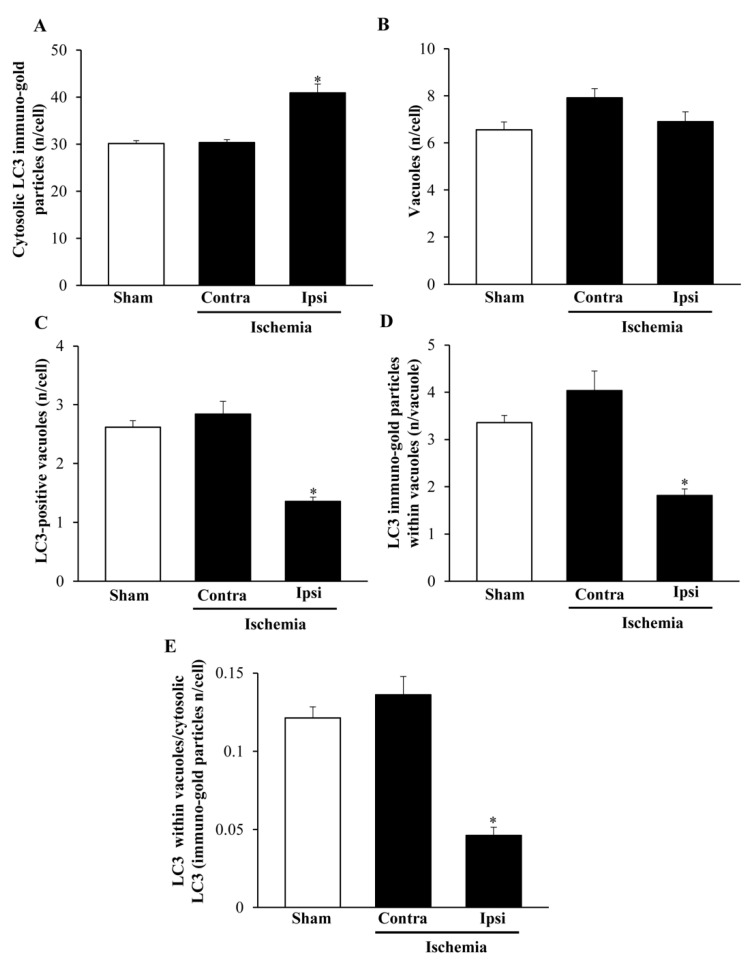
Ultrastructural morphometry of vacuoles and LC3 within ventral *area penumbra*. Graphs report the ultrastructural morphometry for LC3 and vacuoles from ventral *area penumbra* (Ipsi/Ischemia, *n* = 6) and homologous regions from the contralateral side (Contra/Ischemia, *n* = 6) or from sham-operated mice (Sham, *n* = 6). Values were reported as the mean ± S.E.M. per cell from a total of 120 cells for each group. * *p* ≤ 0.05 compared with other groups. (**A**) df = 2, F-value = 24.891, *p* < 0.0001; (**B**) df = 2, F-value = 3.475, *p* = 0.0575; (**C**) df = 2, F-value = 29.307, *p* < 0.0001; (**D**) df = 2, F-value = 20.581, *p* < 0.0001; (**E**) df = 2, F-value = 32.465, *p* < 0.0001.

**Figure 13:**
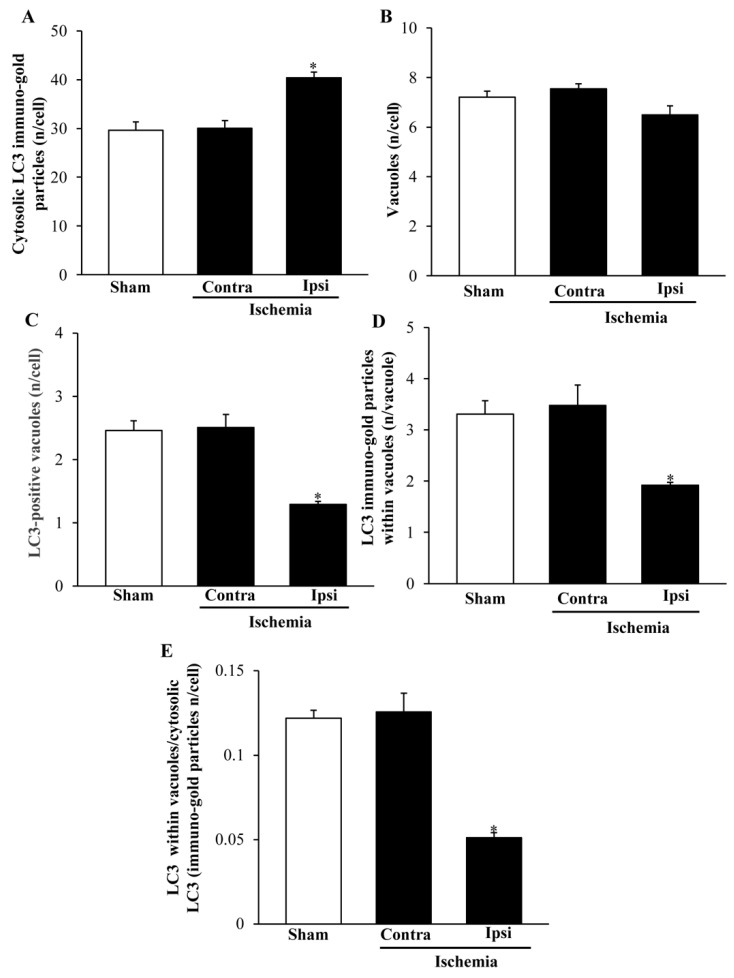
Ultrastructural morphometry of vacuoles and LC3 within dorsal *area penumbra*. Graphs report the ultrastructural morphometry for LC3 and vacuoles from dorsal *area penumbra* (Ipsi/Ischemia, *n* = 6) and homologous regions from the contralateral side (Contra/Ischemia, *n* = 6) or from sham-operated mice (Sham, *n* = 6). Values were reported as the mean ± S.E.M. per cell from a total of 120 cells for each group. * *p* ≤ 0.05 compared with other groups. (**A**) df = 2, F-value = 16.418, *p* = 0.0002; (**B**) df = 2, F-value = 3.779, *p* = 0.0469; (**C**) df = 2, F-value = 20.645, *p* < 0.0001; (**D**) df = 2, F-value = 9.565, *p* = 0.021; (**E**) df = 2, F-value = 33.527, *p* < 0.0001.

**Figure 14:**
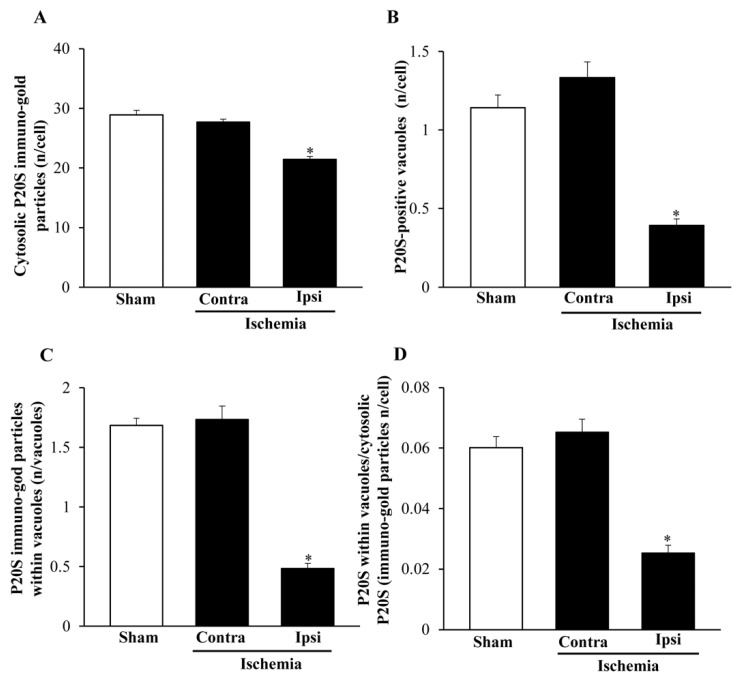
Ultrastructural morphometry P20S within ventral regions. Graphs report the ultrastructural morphometry for P20S from ventral *area penumbra* (Ipsi/Ischemia, *n* = 6) and homologous regions from the contralateral side (Contra/Ischemia, *n* = 6) or from sham-operated mice (Sham, *n* = 6). Values were reported as the mean ± S.E.M. per cell from a total of 120 cells for each group. * *p* ≤ 0.05 compared with other groups. (**A**) df = 2, F-value = 43.562, *p* < 0.0001; (**B**) df = 2, F-value = 40.712, *p* < 0.0001; (**C**) df = 2, F-value = 84.252, *p* < 0.0001; (**D**) df = 2, F-value = 36.64, *p* < 0.0001.

**Figure 15:**
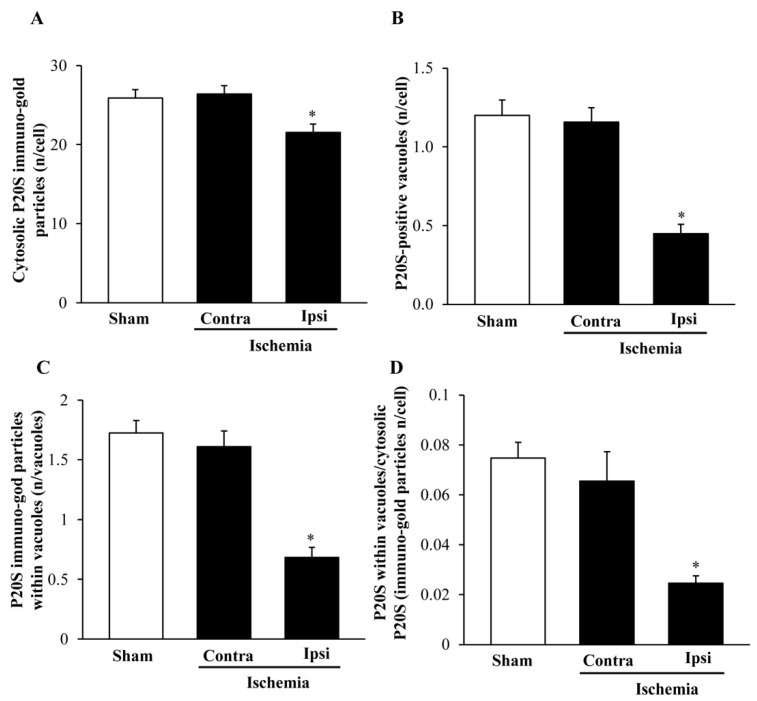
Ultrastructural morphometry of P20S within dorsal regions. Graphs report the ultrastructural morphometry for P20S from dorsal *area penumbra* (Ipsi/Ischemia, *n* = 6) and homologous regions from the contralateral side (Contra/Ischemia, *n* = 6) or from sham-operated mice (Sham, *n* = 6). Values were reported as the mean ± S.E.M. per cell from a total of 120 cells for each group. * *p* ≤ 0.05 compared with other groups. (**A**) df = 2, F-value = 6.403, *p* = 0.098; (**B**) df = 2, F-value = 25.297, *p* < 0.0001; (**C**) df = 2, F-value = 27.547, *p* < 0.0001; (**D**) df = 2, F-value = 11.257, *p* = 0.001.

The occurrence of LC3 (Figure 9), P20S (Figure 10), and their merging (Figure 11) are evident within specific vacuoles which can be detected both within ventral *area penumbra* and within the ventral sham region or within the contralateral homologous area. These three figures reporting representative images indicate the placement of both LC3 and P20S particles within multi-membrane gold standard autophagy vacuoles owing a cytosol-like electron-density as per Autophagy Guidelines [18]. The quantitative stoichiometry of these antigens and autophagy vacuoles as well as the placement of antigens within specific compartments is reported in Graphs of Figure 12, Figure 13, Figure 14 and Figure 15.

When performing electron microscopy, we added as a control the contralateral side, in order to provide a quantification of those potential changes occurring within the non-ischemic side of mice undergoing the mono-lateral (right) irreversible occlusion of MCA. In this way, graphs for quantitative stoichiometry report the ischemic penumbra (Ipsi/Ischemia) along with the homologous region from the contralateral (left) side (Contra/Ischemia). Additionally, homologous regions in the right hemisphere of sham mice (Sham) were included. Such an add on was provided to assess potential changes induced in the whole brain by ischemia. In fact, the fine quantitative assessment provided by ultrastructural stoichiometry allows us to detect changes that are slighter to be assessed by light microscopy. The amount of immunofluorescence indicates a rough variation in the amount of a protein, but it does not tell the real number of the antigen molecules, which instead are detected by immuno-gold-based stoichiometry. Thus, by profiting off single-molecule detection provided by immuno-gold, we compared the contralateral side of ischemic mice with regions homologous to *area penumbra* from sham-operated mice. In this way, systemic effects on these proteins induced by focal ischemia could be disclosed.

The combination of quantitative stoichiometry with plain ultrastructure also allows us to detect the number of vacuoles within various brain regions and the number of proteins within vacuoles compared with cytosol.

### 2.6. The Amount and Compartmentalization of LC3 within Area Penumbra

As reported in graphs of Figure 12 and Figure 13, ultrastructural stoichiometry confirms the increase in LC3 particles within neurons from ventral and dorsal *area penumbra*, respectively. The increase is similar in both regions when measured as the amount of whole cytosol LC3 immuno-gold particles (Figure 12A and Figure 13A). The significance of the present study is focused on the merging within vacuoles of both LC3 and P20S particles. Thus, a measurement of total vacuoles was carried out at first, referring to non-stained, autophagy-like vacuoles. No difference was measured in the amount of these vacuoles within the three different areas: *area penumbra*, the homologous contralateral region, and the homologous area of sham-operated mice. This was replicated within ventral and dorsal regions (Figure 12B and Figure 13B, respectively). Surprisingly enough, when LC3-positive vacuoles (Figure 12C and Figure 13C, ventral and dorsal respectively) and LC3 particles within each vacuole (Figure 12D and Figure 13D, ventral and dorsal respectively) were counted, a dramatic decrease in LC3-stained vacuoles and LC3 particles per vacuole were documented. Such a decrease was roughly half of what counted either in sham-operated or contralateral regions. These results were unexpected based on data from light microscopy and stoichiometric counts of LC3 at electron microscopy in the whole cytosol. In fact, compartmentalization of LC3 within vacuoles, which was measured by the ratio of LC3 within vacuoles vs. LC3 within cytosol was dramatically depressed within *area penumbra* compared with contralateral and sham-operated homologous regions. The loss of LC3 compartmentalization within *area penumbra* was exceeding two-fold both within ventral (Figure 12E) and dorsal (Figure 13E) regions.

### 2.7. The Amount and Compartmentalization of P20S within Area Penumbra

When quantitative stoichiometry of P20S was carried out, similar to light microscopy, a decrease in the amount of cytosol-occurring P20S molecules was detected in the ventral (Figure 14A) and dorsal (Figure 15A) *area penumbra*. Such a decrease was similar within the two *area penumbra*. However, the decrease in P20S was much slighter when the particles were counted at ultrastructural morphometry (a decrease between 10% and 20%) compared with what was measured at light microscopy, where the number of P20S-positive cells was reduced three-fold compared with controls. The stoichiometric counts of P20S were relevant. In fact, when the number of P20S-positive vacuoles (Figure 14B and Figure 15B) or the number of P20S particles within vacuoles (Figure 14C and Figure 15C) were assessed within *area penumbra*, the decrease compared with control was much more relevant compared with the slight difference between *area penumbra* and control, which was measured in the cytosol.

As a consequence, while the decrease of P20S in *area penumbra* compared with control was slight, the concentration of P20S within vacuoles was strikingly depressed within ventral (Figure 14D) and dorsal (Figure 15D) *area penumbra* compared with control.

Thus, by profiting off the stoichiometric count of LC3 and P20S within specific compartments, it was possible to demonstrate that indeed, the discrepancy between LC3 and P20S as measured at light microscopy within *area penumbra* does not quite occur. In fact, just like LC3, a decreased amount of P20S is measured within vacuoles from *area penumbra* neurons. The slight difference between LC3 and P20S, which is measured both at light and electron microscopy concerns only the total cytosolic amount, which is increased (LC3) or slightly decreased (P20S). The striking effect concerns the vacuole compartment where a similar massive decrease was measured for both antigens (LC3 and P20S) within *area penumbra* compared with control tissue.

### 2.8. The Amount and Compartmentalization of P20S + LC3 Vacuoles (Autophagoproteasomes) within Area Penumbra

**Figure 16:**
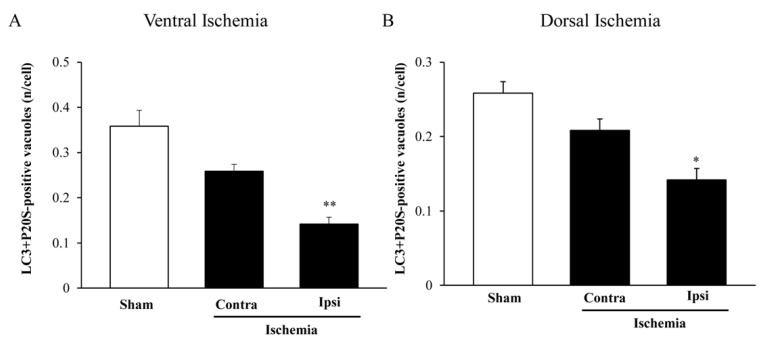
LC3 + P20S vacuoles (autophagoproteasomes) decrease within *area penumbra*. Graphs report the number of LC3 + P20S immuno-gold double-stained vacuoles per cell both in the ventral (**A**) and dorsal (**B**) region. Dorsal or ventral placement of *area penumbra* was dissected in the ipsilateral (right) hemi-encephalon of ischemic mice (Ipsi/Ischemia, *n* = 6), the analogous area was analyzed from the contralateral side (Contra/Ischemia, *n* = 6) and from the ipsilateral hemi-encephalon of sham mice (Sham, *n* = 6). Values are given as the mean ± S.E.M. per cell from a total of 120 cells for each group. * *p* ≤ 0.05 compared with Sham, ** *p* ≤ 0.05 compared with Sham and Contra/Ischemia. (**A**) df = 2, F-value = 20.650, *p* < 0.0001; (**B**) df = 2, F-value = 14.510, *p* = 0.0003.

As shown in Figure 16A (ventral) and Figure 16B (dorsal), the amount of LC3 + P20S positive vacuoles (autophagoproteasomes) is reduced much more compared with the decrease in the merging of LC3 and P20S, when measured at light microscopy. In fact, previously in the text, when commenting on light microscopy data, we wondered how the marked decrease in merging might have occurred to such a massive extent when considering the increase of LC3, which was supposed to mitigate the decrease in P20S. Thus electron microscopy data explain such an apparent discrepancy and provide the mechanistic analysis explaining the massive decrease of autophagoproteasomes within *area penumbra*, which is the concomitant decrease in the amount of both LC3 and P20S from vacuoles. This is independent of the net amount of either LC3 (moderately increased) or P20S (slightly decreased) in the cytosol since the massive decrease in the amount of both antigens from vacuoles prevails at large over the effects measured in the cytosol. It is likely that concomitant measurement of both molecules which are decreased from vacuoles within the peri-infarct region (*area penumbra*) may explain why in this case we measured a difference between homologous areas contralateral to ischemia compared with sham-operated mice. The compartmentalization of both autophagy and proteasome markers within *area penumbra* was confirmed by quantitative ultrastructural morphometry in mice sacrificed at 6 h or 72 h following ischemia (Supplementary Figures S4 and S5).

It is remarkable to notice that similar findings were obtained at 6 h (Supplementary Figures S4 and S5) and 72 h (Supplementary Figures S4 and S5), which recapitulate the data reported at 24 h (Figure 12, Figure 13, Figure 14, Figure 15 and Figure 16). The decrease in the compartmentalization of LC3, P20S and combined LC3 + P20S, may be due either to a reduction in the entry of antigens within the vacuoles or it may be caused by a loss of antigens prom previously filled vacuoles. The steady amount at various time intervals (at 6 h, 24 h, and 72 h) indicate the same decrease in the amount of LC3 and P20S is measured at different time intervals following ischemia. This leaves open the chance of either decrease entry or increased escape of each antigen to/from the vacuoles. What remains positive evidence concerns the ratio between the amount of LC3 within cytosol and vacuoles. It is clear that the redistribution of LC3 in the cytosol compared with vacuoles does occur.

Since the amount of LC3 or 20S in the vacuoles may reflect a loss of antigenicity due to a partial degradation, we also measured Beclin1, another specific marker of autophagy, which co-localizes with P20S [13], in order to decipher better these data. Results reported in Supplementary Figures S6 and S7 report similar findings, even when Beclin 1 detection was combined with P20S.

## 3. Discussion

The common marker routinely used to mark the *area penumbra* corresponds to the chaperone protein HSP70 [19,20,21]. Beyond its role as a marker, this protein is believed to provide an active role in the modulation of the fate of those neurons placed within such a peri-infarct area. In fact, mice knocked out for HSP70 undergo an enlarged infarct following the same arterial occlusion [22,23]. Again, the molecular mechanisms of HSP70 are known to counteract the deleterious effects produced by brain ischemia [24,25]. It appears that HSP70 may represent just the tip of an iceberg since other proteins are recently described to be increased within *area penumbra*, also these proteins are potentially relevant to modulate the fate of neurons placed within this area. Recently, a number of proteins belonging to the autophagy machinery such as LC3 were probed [4,5]. In detail, LC3 was found to be increased within *area penumbra*. Accordingly, and a number of studies report a beneficial effect of autophagy activation, although their significance remains controversial [4,5,6,7,8].

In the present study, we provide light and electron microscopy evidence showing that LC3 increases in the whole cells of *area penumbra*, while electron and light microscopy indicate a decrease in P20S within the same neurons. Such an apparent discrepancy hides the same molecular phenomenon. In fact, when autophagy-like vacuoles are analyzed, a decreased amount of both LC3 and P20S was measured within vacuoles despite LC3 was increased and P20S was decreased in the whole cytosol within *area penumbra* neurons. In keeping with the classic concept, the decreased amount of LC3 *per se* suggests a de-potentiation of the autophagy machinery, which leads to revising the literature showing an increase of LC3 within *area penumbra* cells. This is in line with recent data by Rami et al. (2008) at light microscopy showing redistribution of LC3 within area *penumbra* neurons [6]. This generates puncta that usually are interpreted as vacuoles. Conversely, the authors posed some doubts on the vacuolar nature of these redistributed puncta. In the present manuscript, we provide direct evidence about the nature of LC3 redistribution within *area penumbra* by studying LC3 compartmentalization by quantitative ultrastructural morphometry. In fact, here we measure a decrease in the amount of LC3 within autophagy vacuoles which is redistributed outside the vacuoles towards cytosolic compartments.

This may be due either to a reduction in the entry of LC3 within the vacuoles or it may be caused by a loss of LC3 from previously filled vacuoles. The additional time intervals (shorter at 6 h, and longer at 72 h, compared with the 24 h once reported) indicate that even a few hours after the ischemia, the same decrease in the amount of LC3 is measured and this persists at 3 days. Again, such a decreased amount may be the consequence of a potentiated degradation of LC3 within vacuoles. Such a hypothesis remains to be investigated. Nonetheless, one should consider that a similar loss within autophagosomes is measured for P20S, which markedly reduces the amount of autophagoproteasomes and rules out a proteasomal degradation of LC3. Again, when another autophagy marker such as Beclin1 was measured, its amount was decreased as well in the vacuoles despite an increase in the cytosol just like LC3. Similarly, a decrease in the combined concentration of Beclin1 + P20S within vacuoles was measured in the present study.

Apart from these hypotheses, it remains solid evidence of the present study that, the ratio between the amount of LC3 within vacuoles and cytosol decreases in *area penumbra*, and redistribution of LC3 in the cytosol compared with vacuoles does occur.

In keeping with cell clearing systems, *area penumbra* is also characterized by altered expression of proteasome-related proteins [9]. In fact, both autophagy and proteasome are active in brain ischemia, where an interplay may take place concerning a shift from autophagy to proteasome activity [10] or vice-versa [11]. This is related to compensatory events, which are still in the process to be functionally elucidated [12].

No study so far had investigated the compartmentalization of autophagy and proteasome markers within *area penumbra* and no evidence is available concerning the concomitant expression of LC3 and P20S within *area penumbra* when referring to intracellular compartmentalization within neurons. This is key, especially when considering the role played by these proteins in neuron survival and in the light of recent data showing co-localization of LC3 and P20S within the same vacuoles [13,14,15]. The present data confirm an increased expression of LC3 within *area penumbra,* which mimics what is described following the staining with HSP70. Concomitantly, when the occurrence of P20S is analyzed, *area penumbra* is characterized by a loss of this proteasome antigen. To our knowledge, this is the first evidence showing the sub-cellular compartmentalization of LC3 within *area penumbra* and the first study, which report the occurrence of P20S within *area penumbra* so far. Compared with HSP70 and LC3, the trend of P20S expression which marks *area penumbra* is opposite, i.e., P20S is suppressed within this peri-infarct area. Since LC3 and P20S were recently described to co-localize within the same vacuoles named autophagoproteasome [13,14,15], we eventually analyzed the occurrence of such a merging organelle within *area penumbra*.

Within *area penumbra,* neurons autophagy like vacuoles are similar to control tissue, however, LC3 is decreased within vacuoles. Such a cytosolic redistribution is likely to produce fluorescent puncta, which are due to cytosolic LC3 aggregates. This could also be due to a loss of antigenicity of LC3 within vacuoles. In any case, the autophagy vacuoles are supposed to become defective despite the net amount of LC3 in the cell is increased since they do not store LC3 anymore or LC3 is altered. Similar to LC3, the proteasome component P20S was shown to decrease markedly within vacuoles from *area penumbra* neurons. In contrast to LC3, P20S also decreases in the whole cell from *area penumbra*. Indeed, the decrease of P20S within the whole cell is slight (20%) when compared to its massive decrease from vacuoles (300%). Thus, within *area penumbra* cells, the amount of P20S is reduced from vacuoles similarly to LC3. Similarly, the amount of LC3 is greatly reduced from autophagy vacuoles to an amount, which is in excess compared with its increase within the cytosol. In this way, both LC3 and P20S may no longer be committed to produce their specific cell clearance and protect neurons from ischemia-induced damage.

The loss of autophagoproteasomes, which is severe within *area penumbra* is also slightly detected within the contralateral (left) homologous area of mice undergoing mono-lateral (right) MCA occlusion. This might depend on the powerful stressful conditions, which are produced in the course of a stroke, which is supposed to alter the activity of reticular nuclei, which project directly to the cortex. Recent studies indicate that a marked LC3 redistribution is produced by the neurotransmitter norepinephrine [26], which is abundantly released by reticulo-cortical pathways in stressful and life-threatening conditions.

The evidence recently published [15] about enzymatic activity within LC3 + P20S immune-positive autophagoproteasome, further strengthens this concept. In fact, this organelle is greatly de-potentiated by ischemia. This suggests that, despite autophagy depotentiation and proteasome alterations due to protein dislocation, other clearing compartments might be most affected within *area penumbra*. In keeping with this, when the ultrastructural morphometry of the autophagoproteasome is measured, the suppression of such an organelle appears to be the most impressive within *area penumbra*. In these neurons, the amount of total vacuoles stay steady, while the decrease in total proteasome is slight, and the increase in LC3 is evident. All these changes are slighter compared with the massive decrease in autophagoproteasome, which is documented in the present manuscript. The functional significance of these findings deserves an appropriate extensive and dedicated experimental design. Nonetheless, the functional significance of an increased expression of autophagoproteasome is evident following mTOR inhibition exerted by rapamycin [13]. In these experimental conditions, all the proteosome detectable at confocal microscopy enters within LC3 positive autophagy vacuoles. This generates an empowered catalytic activity, which enhances protein degradation. In fact, in these rapamycin-treated cells, LC3 co-immunoprecipitates with P20S, ubiquitin and the misfolded protein alpha synuclein [13,14,15]. In the same experimental conditions, rapamycin rescues cells from synuclein-dependent toxicity [14]. It is likely that, due to the relevance of misfolded proteins and alpha synuclein, which are deleterious to neuron survival and accumulate within *area penumbra*, a deficiency of autophagoproteasome may be rescued by mTOR inhibitors to improve the outcomes of neurons within *area penumbra* by restoring an effective cell clearance.

The present study is the first report to describe the distribution of LC3 and P20S within *area penumbra* showing a decreased amount of these proteins from their natural (LC3) or putative (P20S) sites (vacuoles), where they are needed to properly exert cell clearing function. The functional significance of these ultrastructural changes remains to be determined and it will take a novel experimental stream to decipher the fine-tuning of this phenomenon.

## 4. Materials and Methods

### 4.1. Permanent Focal Ischemia in Mice

A total of *n* = 38 Adult C57BL/6 male mice (Charles River, Calco, Lecco, Italy) weighing 25 g, were housed under controlled conditions (ambient temperature, 22 °C; humidity, 40%) on a 12 h light-dark cycle with food and water *ad libitum*. The experimental protocol was approved by the Ethical Committee of Neuromed Institute (Pozzilli, Italy) further supervised by The Italian Ministry of Health (Authorization number 1194/2020-PR). The animals were anaesthetized with chloral hydrate (400 mg/kg, i.p.). With the aid of an operating stereomicroscope, an incision was made between the outer canthus of the eye and the external auditory meatus. The temporal muscle was bisected and retracted to expose the temporal (lateral) surface of the skull. The MCA was exposed in its lateral superficial course through a small drill-operated craniotomy (0.5 mm^2^). When drilling, the deepest bone layer was preserved to avoid drill penetration through the dura mater and cortical mechanical and thermal damage. In fact, such a layer was carefully removed manually to reach out to the distal middle cerebral artery (MCA), which was occluded by using electrocoagulation. The arrest of blood flow in the distal segment of MCA was visualized by a stereo-microscope. The site of occlusion corresponds to the centre of the red circle pointed by the red arrow of Figure 1. The temporal muscle and overlying skin incision were sutured by using 5/0 polyglactin sutures [27,28]. Body temperature was monitored during surgery by a rectal probe, which was connected through a feedback system to the surgery pad, where the mouse was nested. This system allows keeping the body temperature steady, at 37 °C. Sham-operated animals undergo the same procedure, but MCA cauterization. Mice are sacrificed at 24 h following surgery when brains were dissected for histological/immune-histochemical analysis.

### 4.2. Tissue Preparation

Brains were dissected out and immediately placed into a Carnoy fixing solution composed of ethyl alcohol (60%), acetic acid (10%), and chloroform (30%) for histochemistry and immuno-histochemistry. Twenty-four hours later, brains were placed into 70% ethanol to be wax-embedded (paraffin). The brains were cut at microtome (Leica Microsystem, RM2125, Milan, Italy) into 10 μm-thick coronal slices, which were regularly spaced 550 µm through the whole rostro-caudal extent of the ischemic region. Sections were used for histological and immuno-histochemical analysis.

### 4.3. Histology

Sections were de-waxed and processed for staining with thionin (a kind of Nissl staining) to assess the localization of stroke-induced brain damage. Further morphological analysis was carried out on 10 μm thick slices, which were regularly spaced every 550 µm, through the extension of the ischemic region. This space interval corresponds to a standardized procedure, which allows us to measure ischemic brain volume based on 10 slices. The infarct area in each stained section appears as reported in Figure 2, the necrotic area was outlined at a magnification of ×2.5 and it was quantified using Scion Image software (NIH, Bethesda, MD, USA) [29].

### 4.4. Immune-Histochemical Analysis

Brain slices were used for LC3, P20S, and gold standard HSP70 immune-staining. Slices were treated with normal sera for 1 h (10% in TBS).

Double immunofluorescence for LC3 and P20S was performed by incubating mouse brain sections overnight with polyclonal rabbit anti LC3B (1:50; Santa Cruz Biotechnology, Dallas, TX, USA), polyclonal mouse anti-P20S (1:100; Abcam; Cambridge, UK), and then for 1 h with secondary CY3-coupled anti-rabbit or Fluorescein anti-mouse IgG (1:100; Vector Laboratories, Burlingame, CA, USA) (Table 1). Control staining was performed without primary antibodies. Nuclear staining was performed with 4′,6-Diamidine-2′-phenylindole dihydrochloride (DAPI).

HSP70 or Caspase 9 active (cleaved) immunoperoxidase was performed by incubating mouse brain sections with normal serum for 1 h (10% TBS). Sections were incubated overnight with mouse anti-HSP70 antibody (1:100; R&D Systems, Minneapolis, MN, USA) or rabbit anti-Caspase 9 antibody (1:50; Millipore, Burlington, MA, USA), and then for 1 h with a secondary biotinylated-anti mouse. Control staining was performed without a primary antibody.

### 4.5. SDS Page Immunoblotting

Brain tissues were homogenized at 4 °C in ice-cold lysis buffer with phosphatase and protease inhibitor. One microliter of homogenates was used for protein determinations (Bradford procedure). Proteins (20 µg) were separated on sodium dodecyl sulphate gels (polyacrylamide PVDF precast gel 4–20% gradients) and transferred on transblot-turbo (Bio-Rad, Milano, Italy) for 7 min mixed molecular weight. Filters were blocked 2 h in Tween-20 Tris-buffered saline (TTBS) (100 mM TrisHCl, 0.9% NaCl, 1% Tween 20, pH 7.4) containing 5% non-fat dry milk. Blots were incubated overnight at 4 °C with the following primary antibodies: rabbit polyclonal anti HSP70 (1:1000; Cell Signaling, Danvers, MA, USA), mouse monoclonal anti LC3B (1:1000; MBL International, Woburn, MA, USA), mouse monoclonal anti P20S (1:1000; Abcam, Cambridge, UK). For the housekeeping, we have used beta actin. Blots were incubated with primary mouse monoclonal anti beta actin antibody (1:25,000, Sigma Aldrich, St. Louis, MO, USA) for 1 h at 22 °C. The filter was washed 3 times with TTBS buffer and then incubated for 1 h with secondary peroxidase-coupled antibodies (anti-mouse, 1:3000; Calbiochem, Milan, Italy). Immunostaining was revealed by enhanced chemiluminescence luminosity (GE Healthcare, Milan, Italy). Densitometric analysis was performed with IMAGEJ software. Data were expressed as the mean ± S.E.M.

### 4.6. Transmission Electron Microscopy (TEM)

Twenty-four hours following ischemia, blocks from the cerebral cortex (1 mm × 1.3 mm × 1 mm) were dissected in the ipsilateral hemi-encephalon of ischemic mice (*n* = 6) and from the contralateral side (*n* = 6) as schematically marked within representative Figure 3. From each side of each mouse, both a ventral and a dorsal border of the infarct area were analyzed. This makes the regions classified as *area penumbra* as a dorsal and ventral *area penumbra* of the side ipsilaterally to ischemia and a similar number of regions in the contralateral non-ischemic side. Therefore, the number of *area penumbra* corresponds to 2 (dorsal and ventral) ipsilateral for each mouse (*n* = 6) undergoing ischemia for a total of 6 × 2 = 12 *area penumbra* (Figure 3). Similarly, 12 corresponding regions were analyzed from the contralateral side of the same mice. In addition, since 6 sham-operated mice were used, each once carrying a bilateral dorsal and ventral region, a total of 24 regions corresponding to the very same placement of *area penumbra* in ischemic mice were counted from 6 sham-operated mice. In detail, when analyzing the infarct zone from the 6 ischemic mice, we dissected both the frankly infarct region (core) and the peri-infarct area (*area penumbra*). This allows a qualitative ultrastructural comparison between *area penumbra* and the core of the ischemic region. 

In addition, two other time intervals, namely 6 h and 72 h following ischemia, were added. In detail, for the analysis at 6 h, *n* = 6 ischemic mice and *n* = 4 sham-operated mice were used. For the analysis at 72 h, *n* = 5 ischemic mice and *n* = 4 sham-operated mice were used. To visualize directly, at a glance, all these samples isolated from the ischemic hemispheres (as well as the corresponding regions from contralateral sides and bilaterally, from sham-operated mice) are reported as red-lined squares in Figure 3. The use of sham-operated mice in addition to the contralateral (non-ischemic side) served as an additional control to rule out systemic and widespread compensatory changes altering protein expression (LC3, P20S and Beclin 1) all over the brain following focal ischemia, which may potentially involve the contralateral hemisphere. In fact, when such a potential phenomenon may occur, even slightly, on the contralateral side, this may dampen the analytical power of measuring ischemia-induced antigen expression compared with control tissue. For TEM analysis, mice were perfused with fixing solution (paraformaldehyde 2.0%, and glutaraldehyde 0.1%, in 0.1 M PBS, pH 7.4), then brains were dissected and immersed in the same solution overnight at 4 °C. After washing in PBS (0.1 M), samples were post-fixed in 1% osmium tetroxide (OsO_4_) for 1 h at 4 °C. Then, the blocks were dehydrated in a serial gradient of ethanol solutions (30%, 50%, 70%, 90% and 95% for 5 min; and 100% for 60 min), and finally, they were embedded in epoxy resin. The concentration of the fixing and post-fixing solutions, along with the use of the epoxy embedding resin, were validated in previous studies performing immuno-gold-based ultrastructural morphometry [30,31]. In fact, the combination of aldehydes, OsO_4_, and epoxy resin allows a minimal epitope covering while preserving sub-cellular architecture [13,32,33]. For identification of peri-ischemic and contralateral area, 1–2 μm thick semithin sections were obtained with a porter Blum MT-1. Semithin sections were stained with 1% toluidine blue and 1% methylene blue in 1% sodium tetraborate [34] and observed under a Nikon Eclipse 80i light microscope (Nikon, Tokyo, Japan) (Figure 4). From these sections, the isosceles trapezoid area was selected on the embedded tissue blocks to be further cut at ultramicrotome (Leica Microsystems, Wetzlar, Germany) to proceed with the ultrastructural analysis.

Ultrathin sections were used both for plain, and post-embedding immuno-gold electron microscopy. Ultrathin slices were stained with uranyl acetate and lead citrate [35], and they were observed at Jeol JEM SX100 electron-microscope (Jeol, Tokyo, Japan).

### 4.7. Post-Embedding Immuno-Gold Microscopy

Ultrathin sections, collected on nickel grids were processed for proteins detection using primary antibodies detailed in Table 1. These experimental settings for electron microscopy allow detailed morphometry of organelles where membrane appears as sharply contrasting, while concomitant detecting immuno-gold-based stoichiometry allow to mark proteins amount and placement within various cell compartments, such as vacuoles and mitochondria. Ultrathin sections were layered on droplets of aqueous sodium metaperiodate (NaIO_4_) for 30 min, at 22 °C to remove OsO_4_. This step is recommended for antigen unmasking, while keeping optimal preservation of cell ultrastructure [32]. After washing in PBS, grids were exposed to drops of blocking solution (10% goat serum and 0.2% saponin in PBS) for 20 min, at 22 °C. Then, grids were further incubated in a humidified chamber, overnight, at 4 °C with either single or combined primary antibodies (LC3, 1:50; P20S, 1:20; or Beclin 1, 1:50) in ice-cold PBS solution containing 1% goat serum and 0.2% saponin. After washing in cold PBS, ultrathin sections were incubated with gold-conjugated secondary antibodies (both 10 nm and 20 nm immuno-gold particles), both diluted 1:20 within a blocking buffer (1% goat serum and 0.2% saponin in PBS) for 1 h, at 22 °C. Counts of immuno-gold particles (10 nm and 20 nm) were carried out at TEM by using the lowest magnification (8000×), still allowing immuno-gold particles to be identified and whole cell organelles to be counted under the same magnification [36,37]. To count immuno-gold particles in cortical neurons, measurements started from a grid square corner to proceed the scanning of the whole section within that grid square [38]. We counted the total number of immuno-gold particles for each primary antibody within the cytoplasm, within the vacuoles, and within mitochondria for each neuron.

Grids were analyzed in order to count immuno-gold particles within 20 cortical neurons for each mouse (*n* = 6) leading to 120 cells from each group.

### 4.8. Statistical Analyses

Neuronal density values (number of neurons/mm^2^) were calculated in brain slices stained with LC3 and/or P20S. Slices spaced 550 microns apart were calculated within each region (*area penumbra*, homologous regions from sham-operated mice). Four slices were counted for each animal. Values for neuronal density (number of neurons/mm^2^) were calculated within a squared area on a computer monitor, ranging in size around 82,500 μm^2^ (330 × 250 μm) Results are given as cell density per mm^2^. Number of positive neurons are given as the mean ± S.E.M. Inferential statistics to compare groups was carried out by using Student’s *t*-test analysis (H_0_, null hypothesis, was rejected when *p* ≤ 0.05).

For western blot experiments, values of optical density were presented as the mean ± S.E.M. Analysis of variance (one-way ANOVA) was used to compare the experimental conditions and Bonferroni test was used to compare the mean values for all groups. The null hypothesis was rejected for *p* ≤ 0.05.

For electron microscopy, assessment of vacuoles and measurement of immunogold particles were carried out according to Lenzi et al. (2016) [13]. For ultrastructural morphometry, the following items were counted: (i) number of LC3 and P20S, per cell; (ii) number of unstained vacuoles per cell; (ii) number of LC3-, P20S-positive vacuoles per cell; (iii) number of LC3 + P20S-positive vacuoles per cell; (iv) number of Beclin 1 + P20S-positive vacuoles per cell. Moreover, values for ultrastructural morphometry were also expressed as a ratio as follows: (i) number of LC3 immuno-gold particles within vacuoles out of the number of cytosolic LC3 immuno-gold; (ii) number of P20S immuno-gold particles within vacuoles out of the number of cytosolic P20S immuno-gold.

For TEM analysis, the counts were carried out by two different observers, who were blind to treatment for each specific tissue blocks. Data are given as the mean ± S.E.M. Data between groups were compared by using one-way ANOVA with Fisher’s Test. Null Hypothesis H_0_ was rejected when *p* < 0.05.

## Figures and Tables

**Table 1:** Primary and secondary antibodies used in the study.

Antibody	Distributor	Catalog Number	RRID	Concentration
Rabbit polyclonal anti LC3B	Santa Cruz Biotechnology, Dallas, TX, USA	Cod. SC28266	AB_2137719	1:50
Monoclonal mouse anti P20S	Abcam, Cambridge, UK	Cod. AB22674	AB_2171376	1:100 l.m. 1:20 e.m. 1:1000 w.b.
Monoclonal mouse anti HSP70	Thermo Fisher Scientific, Waltham, MA, USA	Cod. 33-3800	AB_2533116	1:100
Rabbit polyclonal anti LC3B	Abcam, Cambridge, UK	Cod. AB128025	AB_11143008	1:50
Rabbit polyclonal anti Beclin 1	Abcam, Cambridge, UK	Cod. AB62557	AB_955699	1:50
Rabbit anti Caspase 9, active (cleaved)	Millipore, Burlington, MA, USA	Cod. AB3629	AB_91558	1:50
Alexafluor 488 anti-mouse	Thermo Fisher Scientific, Waltham, MA, USA	Cod. A21202	AB_141607	1:100
Donkey x Rabbit CY3	Millipore, Burlington, MA, USA	Cod. AP182C	AB_92588	1:300
Goat Anti-Rabbit IgG Antibody, 20 nm Gold Conjugated	Bbi solutions, Edinburgh, UK	Cod. EM GAR20/0.25	AB_1769136	1:50
Goat Anti-Mouse IgG Antibody, 10 nm Gold Conjugated	Bbi solutions, Edinburgh, UK	Cod. EM GAR10/0.25	AB_1769128	1:50
Horse Anti-Mouse IgG Antibody (H + L), Biotinylated	Vector Labs	BA-2000-1.5	AB_2313581	1:200
Fluorescein anti-Rabbit	Vector Labs	FI-1000-1.5	AB_2336197	1:100
Rabbit polyclonal anti HSP70	Cell signaling, Danvers, MA, USA	4872S	AB_2279841	1:1000
Mouse monoclonal anti LC3	MBL International, Woburn, MA, USA	M186-3	AB_10897859	1:1000
Mouse anti beta actin	Sigma Aldrich	A1978	AB_476692	1:25,000
Goat Anti-Rabbit	Millipore	401-393	AB_437797	1:3000
	Millipore	401-215	AB_10682749	1:3000

l.m. = light microscopy, e.m. = electron microscopy; w.b. = western blotting.

## Data Availability

The data that supports the findings of this study are available from the corresponding author upon reasonable request.

## Supplementary Materials

The following are available online at https://www.mdpi.com/article/10.3390/ijms221910364/s1.
