# Supplementary material for: The Autophagy-Related Organelle Autophagoproteasome Is Suppressed within Ischemic Penumbra
Source: Int J Mol Sci. 2021 Sep 26;22(19):10364. doi: 10.3390/ijms221910364 (PMC8508911; doi:10.3390/ijms221910364)
Supplement: Supplementary file 1 [file ijms-22-10364-s001.zip › ijms-1350736-supplementary.pdf]

Supplementary Figures

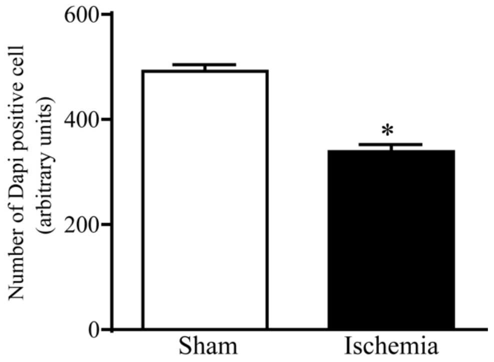

Supplementary Figure S1. Quantitative analysis of Dapi-positive cells.

The graph reports the number of Dapi-positive cell in the ventral *area penumbra* (Ischemia) compared with homologous regions from sham-operated controls (Sham). Graph reports the mean±S.E.M. of Dapi-positive cells. df=19, t=7.877, P< 0.0001.

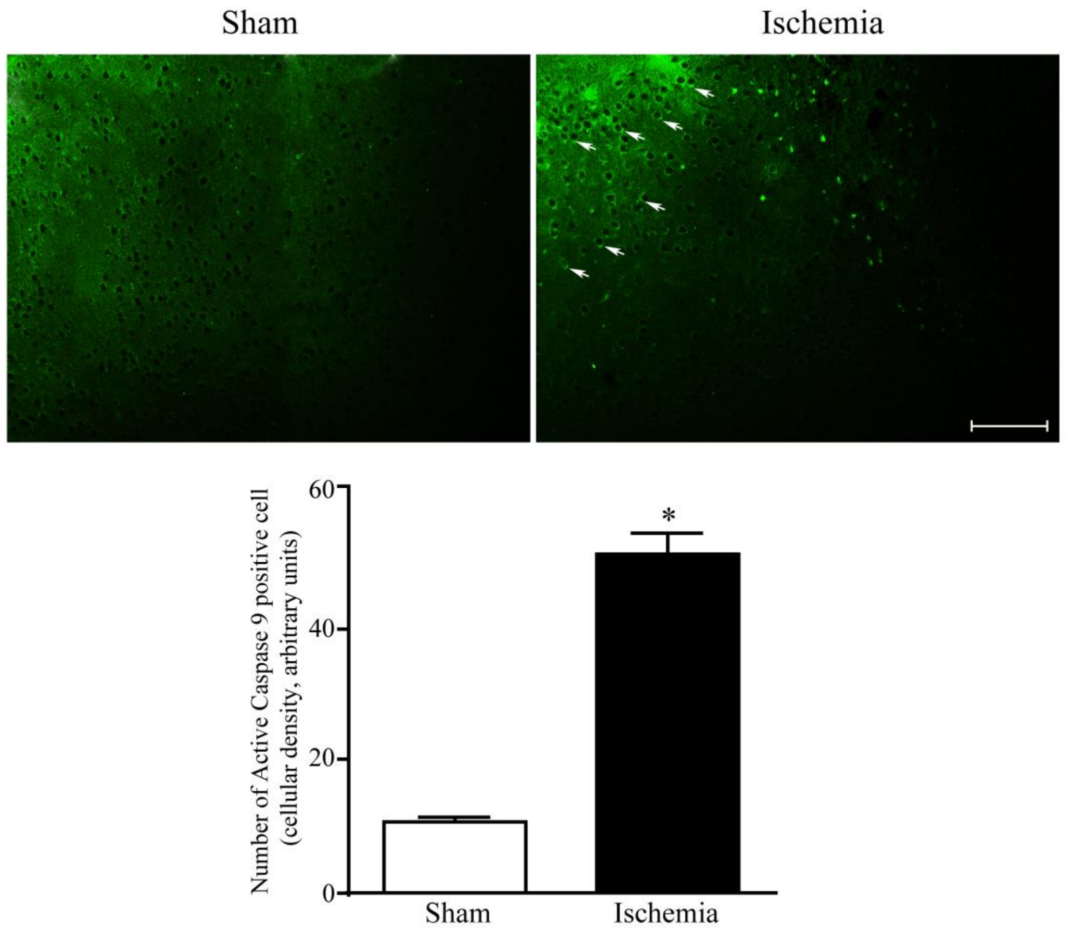

Supplementary Figure S2. Representative Caspase-9-immuno-staining.

This picture shows Caspase-9 positive neurons within the *area penumbra* (Ischemia, in the representative picture) compared with the homologous area from a control mouse (Sham). Arrows point to Caspase-9 immuno-positive neurons. Scale bar=100 µm. df=16, t=12.68, P< 0.0001.

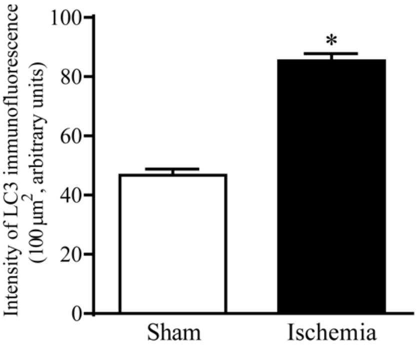

Supplementary Figure S3. Quantitative analysis of LC3 immunofluorescence per 100 µm^2.

The graph reports the quantification of LC3 immunofluorescence normalized to a surface area of 100 µm^2 from homologous regions from sham-operated and ischemia mice. df=40, t=11.97, P < 0.0001.

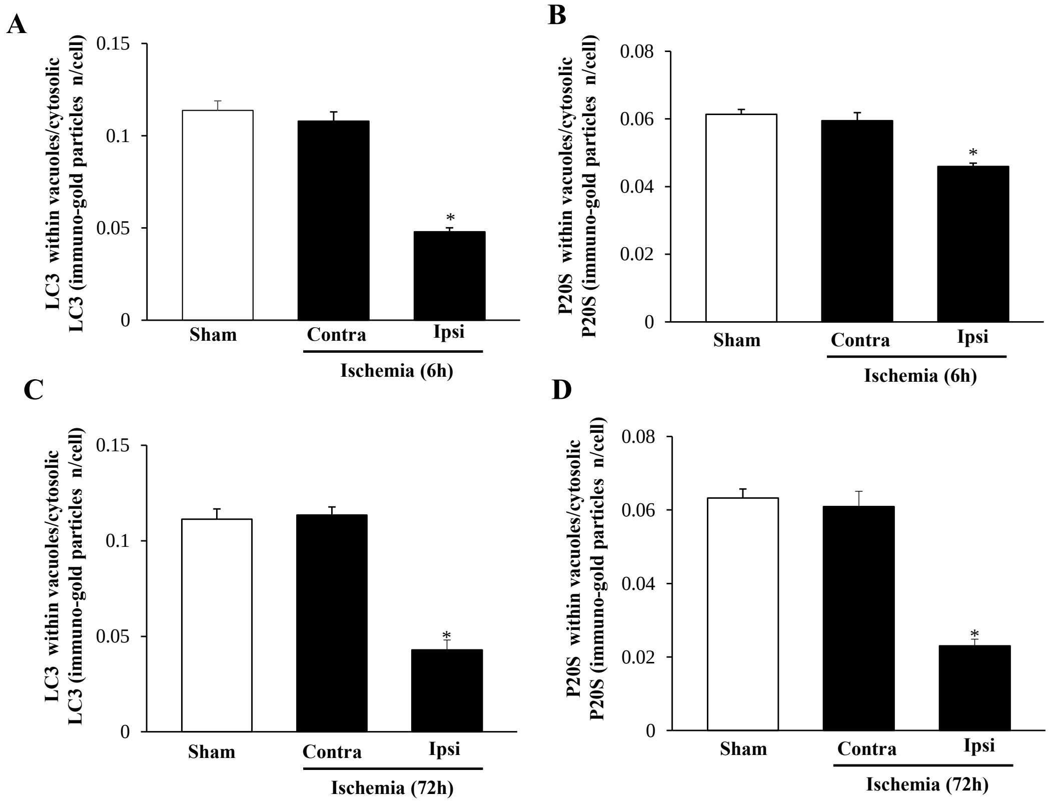

Supplementary Figure S4. Ultrastructural morphometry of LC3 and P20S within ventral regions.

Graphs report the compartmentalization of LC3 (A, C) and P20S (B, D) within ventral regions at 6 hours and 72 hours following ischemia. Values are given as the mean±S.E.M. *P ≤ 0.05 compared with Sham and Contra/Ischemia. (A) df=2, f=75.967, P<0.0001; (B) df=2, f=23.053, P<0.0001; (C) df=2, F=70.328, P<0.0001; (D) df=2, F=55.497, P<0.0001.

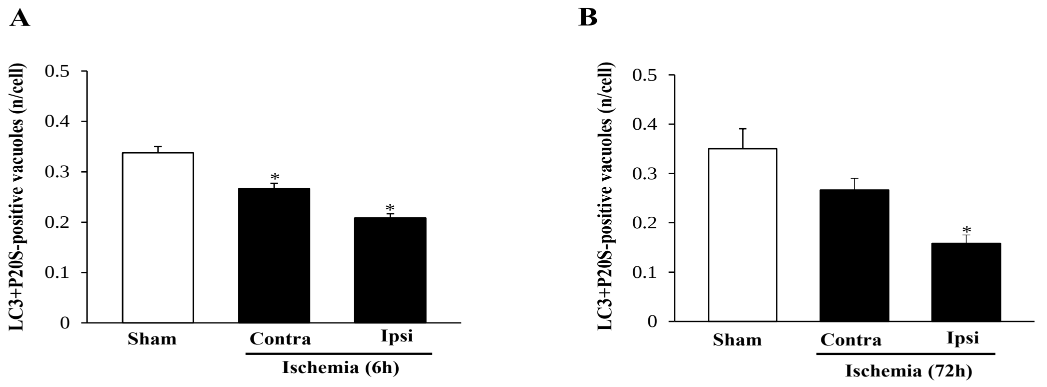

Supplementary Figure S5. LC3+P20S vacuoles (autophagoproteasomes) decrease within *area penumbra* (at 6h and 72h following ischemia).

Graphs report the number of LC3+P20S immuno-gold double-stained vacuoles per cell in ventral region both at 6 hours (A) and 72 hours (B) following ischemia. Analogous area was analyzed from the contralateral side (Contra/Ischemia) and from the ipsilateral hemi-encephalon of sham mice (Sham). Values are given as the mean±S.E.M. *P ≤ 0.05 compared with Sham and Contra/Ischemia. (A) df=2, F=35.843, P>0.0001; (B) df=2, F=13.316, P=0.0012.

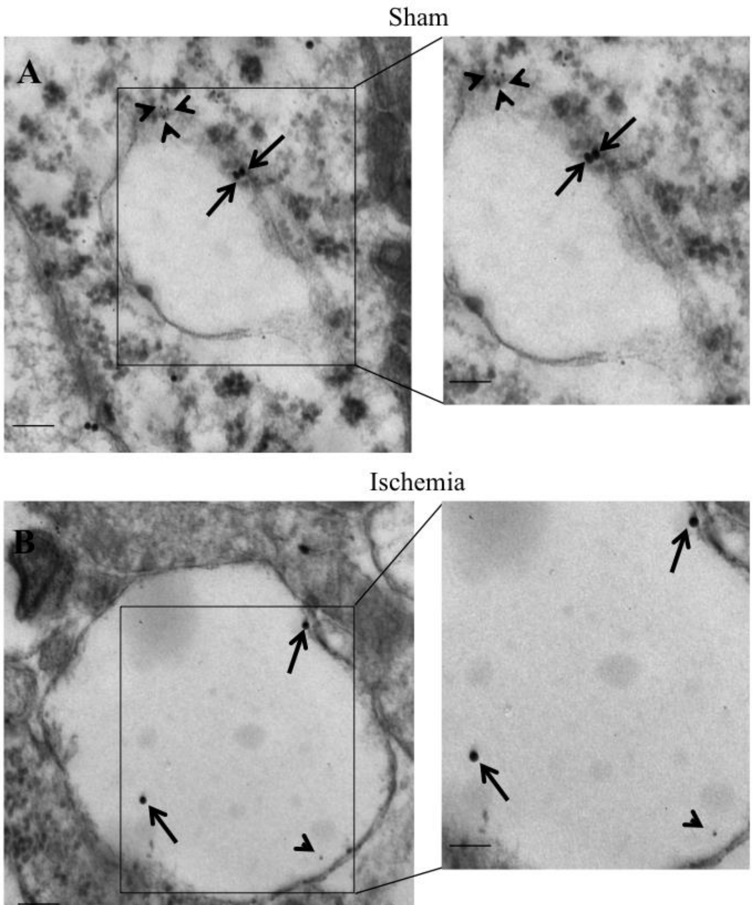

Supplementary Figure S6. Beclin1+P20S co-immuno-stained vacuoles.

Representative TEM micrographs showing Beclin1+P20S- co-immuno-stained vacuoles within a cortical neuron from a sham-operated mouse (A) and a cortical neuron from ventral *area penumbra* from an ischemic mouse (B). Arrowheads

and arrows point to Beclin1 (20 nm) and P20S (10 nm) immuno-gold particles within vacuoles, respectively. The insert highlights the Beclin1+P20S immuno-gold particles within a vacuole. Scale bar= 0.2 μm (A-B), 0.1 μm (inserts).

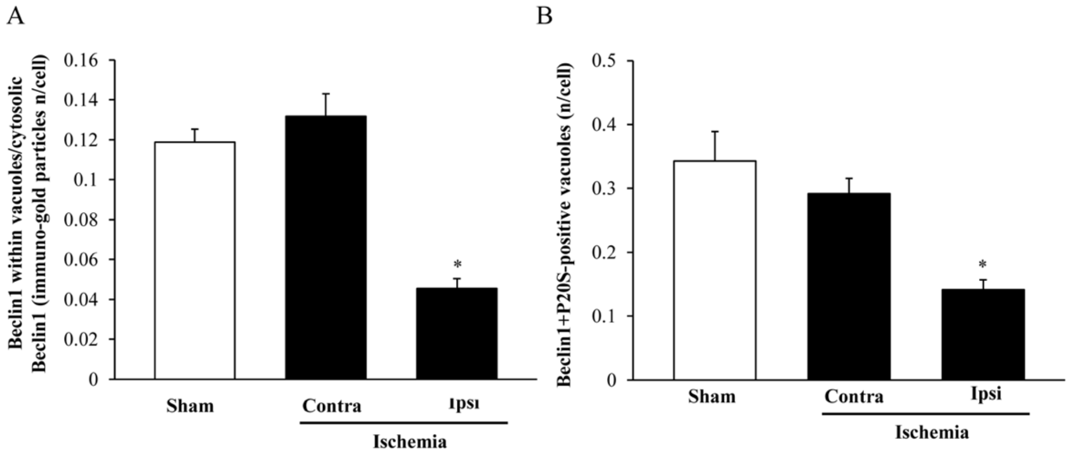

Supplementary Figure S7. Ultrastructural morphometry of Beclin1 within ventral regions.

Graphs report the compartmentalization of Beclin1 (A) and the number of Beclin1+P20S immuno-gold double-stained vacuoles per cell (B) within ventral regions at 24 hours following ischemia. Values are given as the mean±S.E.M. per cell from a total of 120 cells for each group. *$P \leq 0.05$ compared with Sham and Contra/Ischemia. (A) df=2, f=33.336, $P<0.0001$; (B) df=2, f=11.157, P=0.0011.
